# Hsa_circ_0042823 accelerates cancer progression *via* miR-877-5p/FOXM1 axis in laryngeal squamous cell carcinoma

**DOI:** 10.1080/07853890.2021.1934725

**Published:** 2021-06-14

**Authors:** Tianyi Wu, Yanan Sun, Zhanwei Sun, Shichao Li, Weiwei Wang, Boyu Yu, Guangke Wang

**Affiliations:** a Department of Otolaryngology, Head and Neck Surgery, Henan Provincial People’s Hospital, People’s Hospital of Zhengzhou University; People’s Hospital of Henan University, Zhengzhou, PR China; b Department of Otolaryngology, Head and Neck Surgery, The Second Affiliated Hospital, Harbin Medical University, Harbin, PR China

**Keywords:** Circular RNA, hsa_circ_0042823, miR-877-5p, FOXM1, proliferation, migration, invasion, tumour growth, laryngeal squamous cell carcinoma

## Abstract

**Background:**

Laryngeal squamous cell carcinoma (LSCC) is a common malignant tumour of the head and neck. Our previous study reveals that the circular RNA (circRNA) hsa_circ_0042823 is abnormally expressed in LSCC, suggesting that hsa_circ_0042823 is closely associated with LSCC. Here, we attempted to explore the molecular mechanism of hsa_circ_0042823 in LSCC.

**Methods:**

Quantitative real-time PCR and western blot were performed to assess the expression of gene and protein in human laryngeal carcinoma cells, TU212 and TU686. MTT and transwell assays were performed to examine cell proliferation, migration and invasion. The relationship among hsa_circ_0042823, miR-877-5p and forkhead box M1 (FOXM1) was verified by luciferase reporter assay. Finally, we constructed a subcutaneous tumour mouse model to analyse *in vivo* growth of LSCC cells following knockdown of hsa_circ_0042823.

**Results:**

Compared with normal human bronchial epithelial cells (HBECs), hsa_circ_0042823 was highly expressed in the LSCC cell lines (AMC-HN-8 and TU686). Further studies demonstrated that hsa_circ_0042823 interacted with miR-877-5p, and FOXM1 was the target of miR-877-5p. Hsa_circ_0042823 promoted the expression of FOXM1 *via* its ceRNA activity on miR-877-5p. Hsa_circ_0042823 overexpression promoted proliferation, migration and invasion of AMC-HN-8 cells through regulating miR-877-5p/FOXM1 axis. Additionally, inhibition of hsa_circ_0042823 inhibited growth of LSCC *in vivo via* miR-877-5p/FOXM1 axis.

**Conclusions:**

Hsa_circ_0042823/miR-877-5p/FOXM1 axis participates in the progression of LSCC. This work demonstrates that hsa_circ_0042823 accelerates cancer progression by regulating miR-877-5p/FOXM1 axis in LSCC. Therefore, this study may provide new insights into the pathogenesis of LSCC.

**KEY MESSAGES**
- Hsa_circ_0042823 promotes FOXM1 expression by sponging miR-877-5p.
- Hsa_circ_0042823 promotes proliferation, migration, invasion of LSCC cells.
- Hsa_circ_0042823 knockdown inhibits tumour growth of LSCC *via* miR-877-5p/FOXM1 axis.

## Introduction

Laryngeal cancer is a common malignant tumour originated from respiratory system, most of which are laryngeal squamous cell carcinoma (LSCC) [1]. More than 150,000 cases of LSCC are diagnosed every year [2]. In the present society, the smoking rate remains high, and environmental pollution caused by the accelerating of industrialization is becoming more and more serious. Thus, the incidence and mortality of laryngeal cancer are not optimistic. Seeking the specific and effective tumour markers for LSCC has become one of the hot spots in LSCC research.

Circular RNA (circRNA) is an endogenous non-coding RNA that has a closed circular structure. In mammalian cells, exogenous circRNA that can encode proteins has been detected, such as RNA derived from Hepatitis D virus (HDV) [3]. The circRNA of HDV can express and produce pathogenic viral proteins [4]. The vast majority of circRNAs does not encode proteins, but regulate a variety of life activities at the transcription or post-transcriptional level. CircRNA is considered as a novel type of biomarker and therapeutic target for various cancers [5]. Fan et al. have confirmed the abnormal expression of circRNA in LSCC [6]. For example, circRNA CDR1as is up-regulated in tumour tissues of LSCC patients. CDR1as overexpression accelerates the progression of LSCC through miR-7/CCNE1/PIK3CD axis [7]. Up-regulation of circFLNA in tumour tissues of LSCC promotes cell migration by enhancing FLNA and MMP2 expression in LSCC [8]. LSCC tumour tissues exhibit a decrease of hsa_circ_0042666 expression. Hsa_circ_0042666 inhibits LSCC progression by regulating miR-223/TGFBR3 axis [9]. These results have confirmed the vital role of circRNA in the LSCC development. Our previous study has used CeneChip technology to screen out multiple abnormally expressed circRNAs from tumour tissues of LSCC patients, among which hsa_circ_0042823 is abnormally expressed in the LSCC tumour [10]. However, whether hsa_circ_0042823 can regulate LSCC development is still unclear. Thus, we attempted to investigate the function of hsa_circ_0042823 in LSCC.

We used 3 online prediction tools (Circbank, Circinteractome and Starbase V3) to predict the miRNA that hsa_circ_0042823 may bind, showing that hsa_circ_0042823 may interact with hsa-miR-877-5p. Thus, we speculated that hsa_circ_0042823 may play a role in LSCC through binding to miR-877-5p. Furthermore, miR-877-5p is a tumour suppressor and miR-877-5p takes part in the regulation of various cancers such as colorectal cancer and hepatocellular carcinoma [11, 12]. Huang et al. have demonstrated that miR-877-5p targets the transcription factor forkhead box M1 (FOXM1) [13]. Software prediction also revealed that FOXM1 may be served as a downstream target of miR-877-5p. In addition, previous researches have reported that FOXM1 is up-regulated in LSCC [14, 15]. Thus, we speculated that hsa_circ_0042823 may act as a ceRNA of miR-877-5p, thereby promoting the expression of its endogenous target, FOXM1. Hsa_circ_0042823 may promote proliferation, migration, invasion and tumour growth in LSCC through miR-877-5p/FOXM1 axis.

## Materials and methods

### Cell culture

Human bronchial epithelial cells (HBECs) were purchased from ATCC (Cat: PCS-300-013, Manassas, VA). Human laryngeal carcinoma cells, AMC-HN-8 (Cat: BFN60808789) and TU686 (Cat: BFN608007264), were obtained from BLUEFBIO (Shanghai, China).These cells were cultured in Dulbecco’s modified eagle medium (DMEM) (Solarbio, Beijing, China) in a constant temperature incubator of 37 °C and 5% CO_2_. The media were contained 10% foetal bovine serum (FBS, Thermo Fisher Scientific, Waltham, MA) and 1% penicillin/streptomycin (Solarbio).

### Cell transfection

For overexpression of hsa_circ_0042823, the vector pcDNA3.1-containing hsa_circ_0042823 (pcDNA3.1-hsa_circ_0042823) was constructed by GenePharma (Shanghai, China). The pcDNA3.1-NC was used as a negative control (NC).Hsa_circ_0042823-specific small interference RNA (siRNA) (Si-hsa_circ_0042823) and FOXM1-specific siRNA (Si-FOXM1) were used to knock down hsa_circ_0042823 or FOXM1 (GenePharma). Non-silencing siRNA (Si-NC) oligonucleotide was used as a control (Ctrl). MiR-877-5p mimic, mimic NC, miR-877-5p inhibitor and inhibitor NC were synthesized by GenePharma. Lentivector-mediated siRNA hsa_circ_0042823 (LV-si-hsa_circ_0042823) and non-targeting plasmids (LV-Ctrl) were designed and generated by GenePharma. The vectors and oligonucleotides were transfected into cells using Lipo2000 Transfection Reagent (Invitrogen, Carlsbad, CA).

### Quantitative real-time PCR (qRT-PCR)

Extraction of total RNA was carried out using TRIzol reagent (Invitrogen, Carlsbad, CA) . Reverse transcription and PCR reaction were performed applying TaqMan™ Reverse Transcription Reagents (Thermo Fisher Scientific, Waltham, MA) and TB Green^®^ Premix Ex Taq™ II (TliRNaseH Plus) (Takara, Mountain View, CA). Hsa_circ_0042823 was normalized to 18S rRNA, and miR-877-5p was normalized to U6. β-actin was used as a reference gene for mRNA. Data were calculated using 2^−ΔΔCT^ method for quantification.

### Cell proliferation

MTT Cell Proliferation and Cytotoxicity Assay Kit obtained from Beyotime (Shanghai, China) was used to examine cell proliferation. In brief, 100 μL of cells were mixed with MTT reagent (10 µL, 5 mg/mL) and incubated at an atmosphere of 37 °C for 4 h. Then, cells were incubated with Formazan at 37 °C for 4 h. The absorbance of each well was detected at 570 nm using a microplate reader (Thermo Fisher Scientific).

### Cell migration and invasion

Cell migration and invasion were examined using a 24-well Transwell insert system (Corning, NY). For cell migration assay, cells (1.5 × 10^6^ cells/mL) were cultured in the upper chamber-containing FBS-free DMEM at 37 °C with 5% CO_2_ for 24 h. The lower chamber was supplemented with DMEM and 10% FBS. The migrating cells on the below side of chamber were collected. After 95% alcohol of fixation, the migrated cells were stained with 1% crystal violet (Solarbio) for 5 min. Finally, five random visual fields were selected to observe and analyse the differences among groups under an inverted microscope (Olympus, Tokyo, Japan).

For cell invasion assay, matrigel (Becton Dickinson Biosciences, San Diego, CA,) was covered on the basolateral Transwell chambers. Except for this step, cell invasion assay had the same steps with cell migration assay.

### Luciferase reporter assay

The hsa_circ_0042823 and 3′ untranslated region (UTR) of FOXM1 containing the putative binding sites of miR-877-5p were cloned into the vector pGL3, pGL3-hsa_circ_0042823-WT (wild-type, WT), pGL3-hsa_circ_0042823-Mut (or mutant type, Mut), pGL3-FOXM1-WT, pGL3-FOXM1-Mut (GenePharma). WT/Mut of hsa_circ_0042823 or 3′ UTR of FOXM1 vector combined with miR-921 mimic or mimic NC were co-transfected into 293 cells (ATCC, Cat: CRL-1573). The luciferase activity of the transfected 293 cells was detected using the luciferase assay system (Promega, Madison, WI) after 48 h of transfection.

### Western blot (WB)

Total protein extraction was performed applying Total Protein Extraction Kit (Solarbio). Protein concentration was measured using the BCA Protein Assay Kit (Solarbio). Protein samples (20 μg) were separated by 10% SDS-PAGE gel electrophoresis, and then transferred onto the nitrocellulose membrane (Whatman, Maidstone, Kent, UK). The membranes were incubated with rabbit anti-FOXM1 (1:1000, Proteintech, Wuhan, China) or rabbit anti-β-actin (1:5000, Proteintech) at 4 °C for 12 h. The membranes were then treated with HRP-IgG antibody (1:5000, Proteintech). Image J software was used to analyse the data.

### Tumour xenograft experiments

Male BALB/c nude mice (SLAC, Shanghai, China) aged 4–6 weeks were maintained under a specific pathogen-free condition. AMC-HN-8 cells were transfected with LV-Si-hsa_circ_0042823 or LV-Ctrl. Subsequently, BALB/c nude mice were injected subcutaneously with LV-Si-hsa_circ_0042823, LV-Ctrl cells or normal AMC-HN-8 cells (1 × 10^7^ cells/200 μL). The tumour volume was assessed using a vernier calliper. Tumour volume (cm^3^) = ab^2^/2, a: long diameter (cm), b: short diameter (cm). On the 30th day of inoculation, all mice were anaesthetized with 1% pentobarbital sodium (100 mg/kg) by intraperitoneal injection, and then sacrificed by cervical dislocation. The tumour tissues were stripped from the mice, and the tumour weight was detected. All protocols were authorized by the Ethics Committee of Henan Provincial People’s Hospital.

### Statistical analysis

All experiments were performed in triplicate and representative experiments were presented. Data were reported as mean ± SD. Statistical analysis was performed using SPSS 22.0 statistical software (IBM, Armonk, NY). Two-tailed Student’s t, one-way ANOVA was used to analyse the statistical difference. *p* (Two-sided) less than .05 was considered to indicate statistical significance.

## Results

### Hsa_circ_0042823 was up-regulated in AMC-HN-8 and TU686 cells

**Figure 1.**
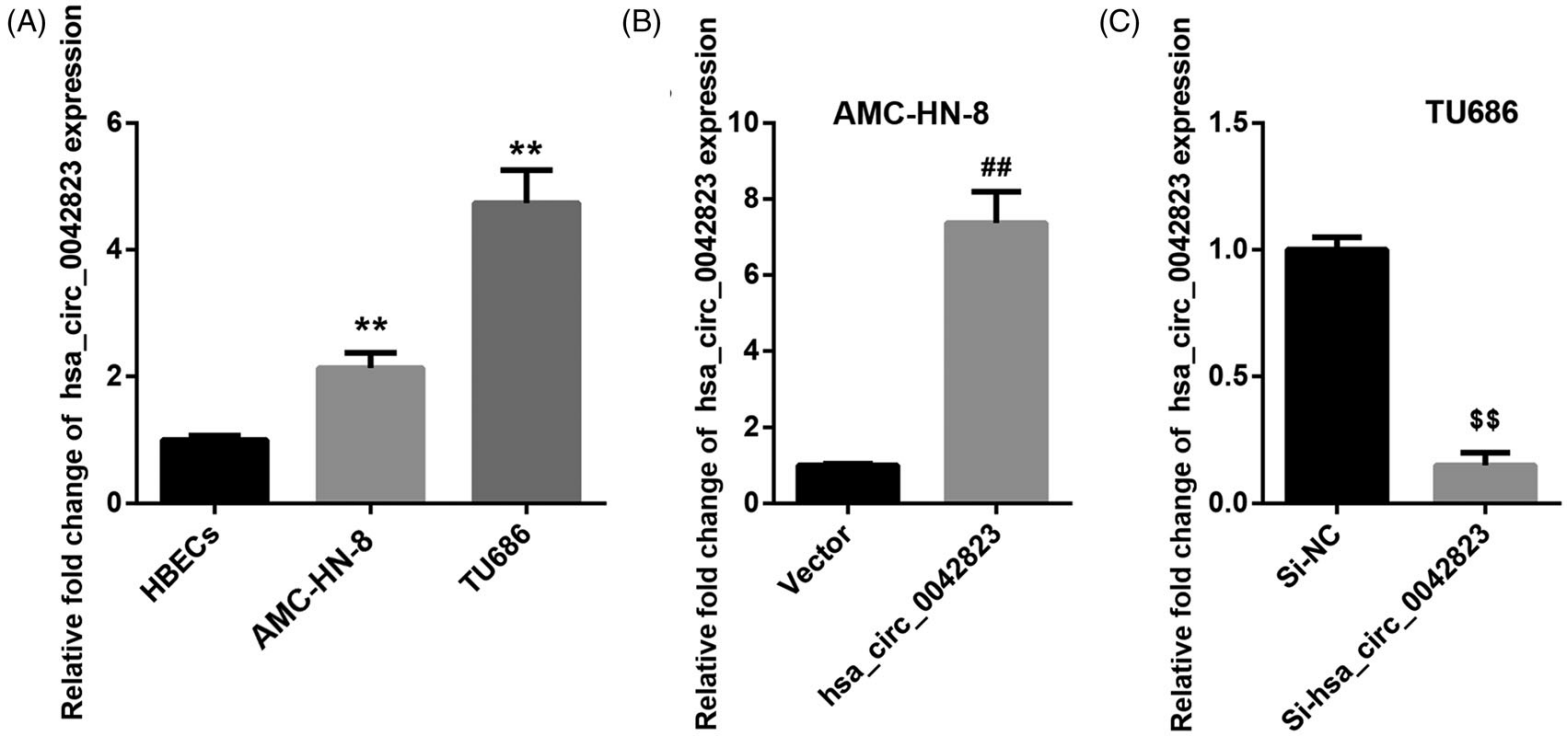
Hsa_circ_0042823 was up-regulated in AMC-HN-8 and TU686 cells. (A) The expression of hsa_circ_0042823 in HBECs, AMC-HN-8 and TU686 cells was assessed through qRT-PCR. (B and C) The qRT-PCR was performed to detect hsa_circ_00428233 expression in the AMC-HN-8 and TU686 cells after hsa_circ_0042823 overexpression or knockdown. **p < .01 compared with HBECs; ^##^*p* < .01 compared with Vector; ^$$^*p* < .01 compared with Si-NC.

To disclose the involvement of hsa_circ_0042823 in LSCC, we compared hsa_circ_0042823 expression between normal HBECs and human laryngeal carcinoma cell lines by qRT-PCR. As shown in Figure 1(A), AMC-HN-8 and TU686 cells displayed an up-regulation of hsa_circ_0042823 with respect to normal HBECs. Then, hsa_circ_0042823 was overexpressed in the AMC-HN-8 cells that express relatively lower expression of hsa_circ_0042823. The results of qRT-PCR revealed that hsa_circ_0042823 expression was notably enhanced in AMC-HN-8 cells after transfected with pcDNA3.1-hsa_circ_0042823 (Figure 1(B)). Furthermore, hsa_circ_0042823 was silenced in TU686 cells that express relatively higher expression of hsa_circ_0042823. In the presence of Si-hsa_circ_0042823, the expression of hsa_circ_0042823 was severely reduced in TU686 cells. The results implied that hsa_circ_0042823 was highly expressed in AMC-HN-8 and TU686 cells.

### Hsa_circ_0042823 overexpression promoted proliferation, migration and invasion of AMC-HN-8 and TU686 cells

**Figure 2.**
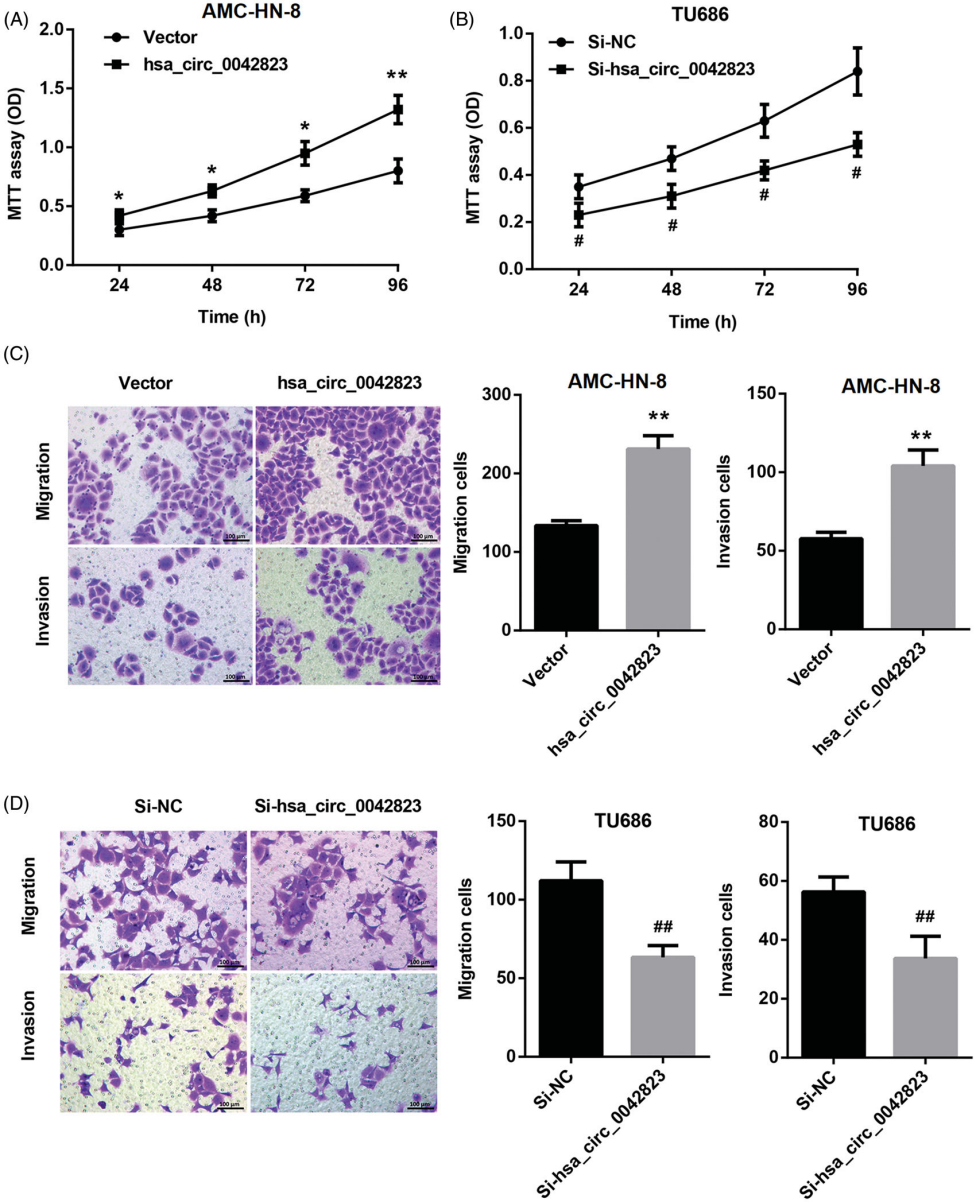
Hsa_circ_0042823 overexpression promoted proliferation, migration and invasion of human laryngeal carcinoma cells. (A and B) MTT assay was performed to examine proliferation of the AMC-HN-8 and TU686 cells after hsa_circ_0042823 overexpression or knockdown. (C and D) Transwell assay was performed to detect migration and invasion of the AMC-HN-8 and TU686 cells after hsa_circ_0042823 overexpression or knockdown. *p < .05, **p < .01 compared with Vector; ^#^*p* < .05, ^##^*p* < .01 compared with Si-NC.

We further investigated the function role of hsa_circ_0042823 in LSCC. The growth curves obtained from MTT proliferation assay revealed that hsa_circ_0042823 overexpression significantly promoted proliferation of AMC-HN-8 cells (Figure 2(A)). The ability of cell proliferation in TU686 cells was severely repressed by hsa_circ_0042823 knockdown (Figure 2(B)). Furthermore, we assessed cell migration and invasion by performing transwell assay. Hsa_circ_0042823 up-regulation caused an increase in migration and invasion of AMC-HN-8 cells (Figure 2(C)). However, the ability of migration and invasion in TU686 cell was significantly decreased following transfection of Si-hsa_circ_0042823 (Figure 2(D)). Therefore, these results suggested that hsa_circ_0042823 had a promoting effect on proliferation, migration and invasion in AMC-HN-8 and TU686 cells.

### Hsa_circ_0042823 enhanced FOXM1 expression via competitively binding miR-877-5p

**Figure 3.**
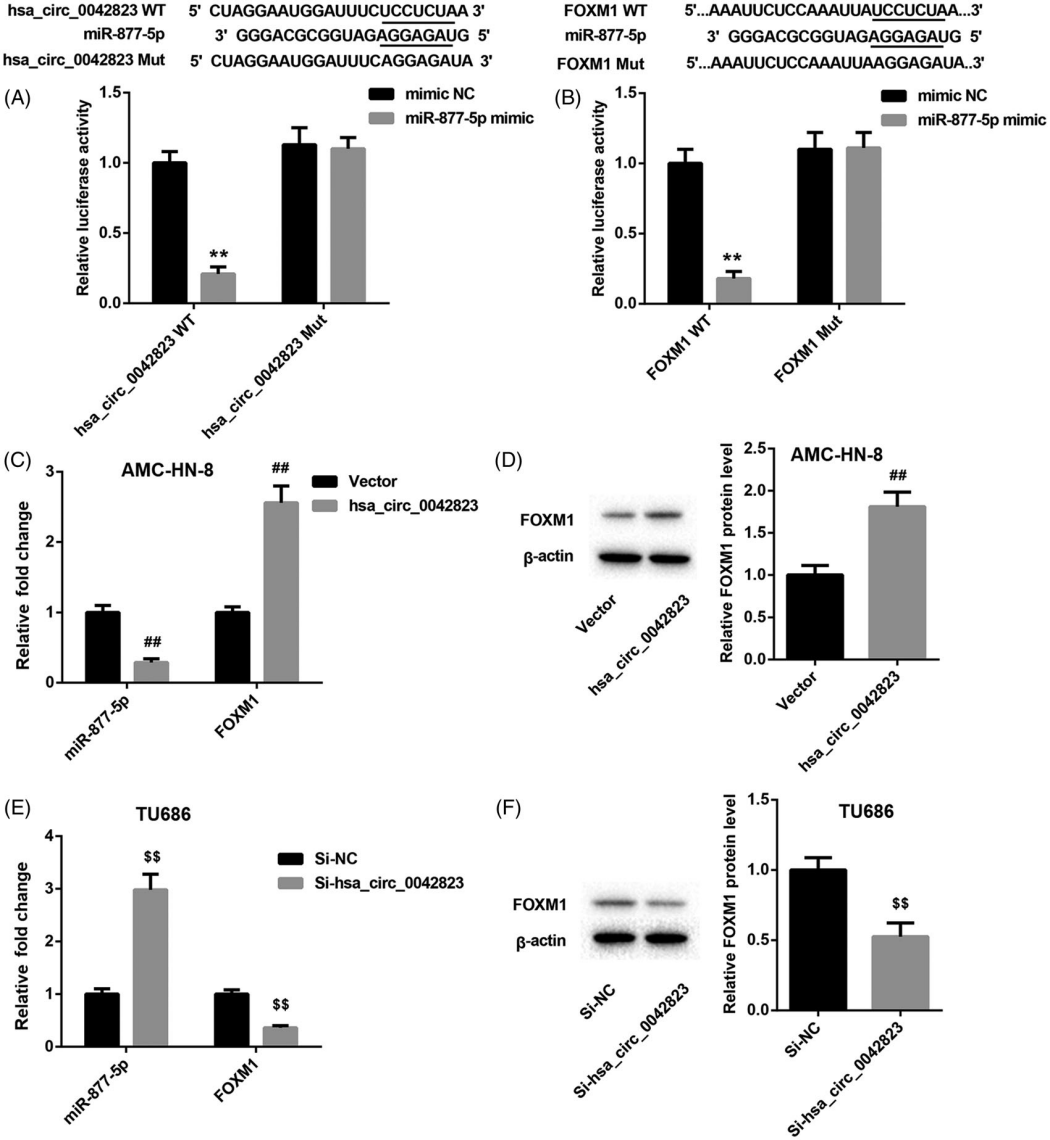
Hsa_circ_0042823 promoted FOXM1 expression by sponging miR-877-5p. (A and B) The relationship among hsa_circ_0042823, miR-877-5p and FOXM1 was verified through luciferase assay. (C and D) The mRNA and protein expression of miR-877-5p and FOXM1 in the AMC-HN-8 cells following hsa_circ_0042823 overexpression was estimated by qRT-PCR and WB. (E and F) The mRNA and protein expression of miR-877-5p and FOXM1 in the TU686 cells following hsa_circ_0042823 knockdown was estimated by qRT-PCR and WB. **p < .01 compared with mimic NC; ^##^*p* < .01 compared with Vector; ^$$^*p* < .01 compared with Si-NC.

The prediction from the database showed that hsa_circ_0042823 may interact with miR-877-5p. To verify this prediction, we performed luciferase activity assay, showing that miR-877-5p was a downstream target of hsa_circ_0042823. Moreover, miR-877-5p interacted with the 3′ UTR of FOXM1 (Figure 3(A,B)). Subsequently, we estimated the regulation mechanism of gene expression among these 3 molecules. Figure 3(C) revealed that the mRNA expression of miR-877-5p was greatly repressed in the AMC-HN-8 cells in the presence of pcDNA3.1-hsa_circ_0042823 (Figure 3(C)). Hsa_circ_0042823 overexpression led to an increase in the mRNA and protein expression of FOXM1 in AMC-HN-8 cells (Figure 3(C,D)). Furthermore, knockdown of hsa_circ_0042823 enhanced the expression of miR-877-5p in TU686 cells, while hsa_circ_0042823 deficiency severely inhibited the mRNA and protein expression of FOXM1 in TU686 cells (Figure 3(E,F)).

**Figure 4.**
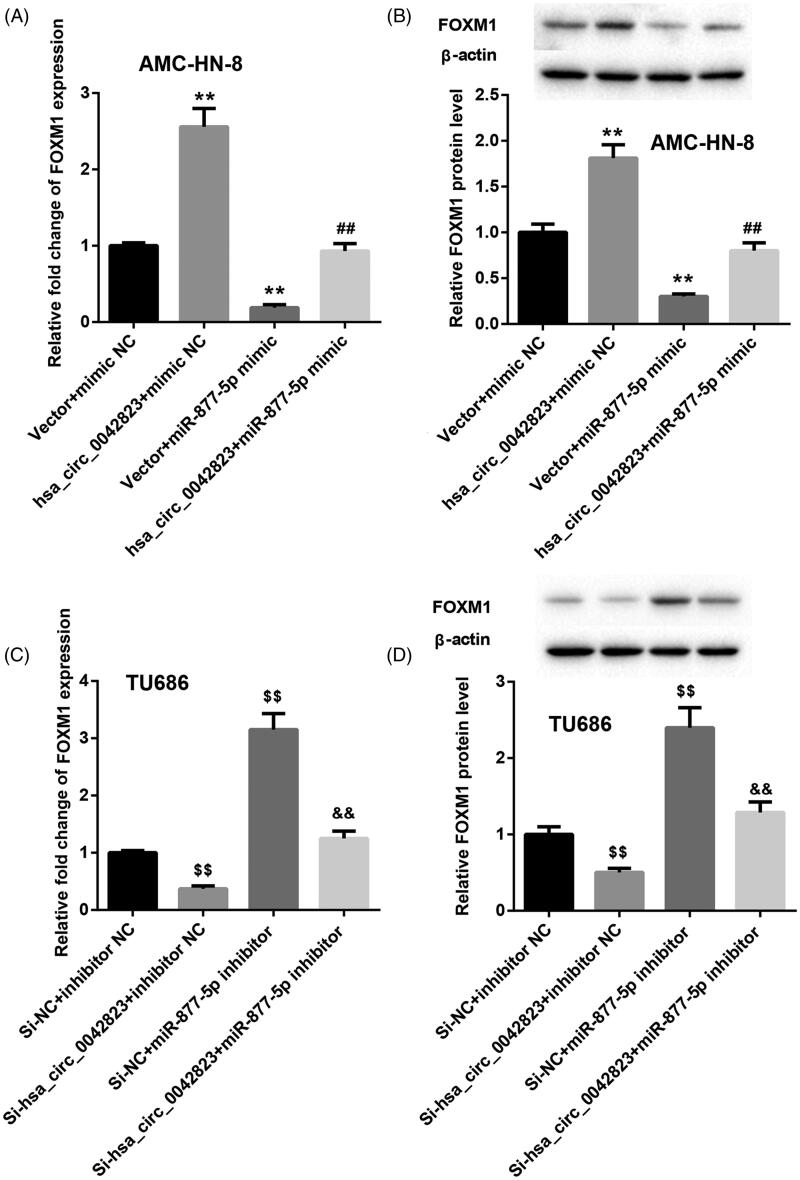
Hsa_circ_0042823 enhanced FOXM1 expression by inhibiting miR-877-5p. (A and B) The mRNA and protein expression of FOXM1 in the AMC-HN-8 cells following up-regulation of hsa_circ_0042823 combined with miR-877-5p was estimated through qRT-PCR and WB. (C and D) The mRNA and protein expression of FOXM1 in the TU686 cells following knockdown of hsa_circ_0042823 combined with miR-877-5p was estimated through qRT-PCR and WB. **p < .01 compared with Vector + mimic NC; ^##^*p* < .01 compared with Vector + miR-877-5p mimic; ^$$^*p* < .01 compared with Si-NC + inhibitor NC; ^&&^*p* < .01 compared with Si-NC + miR-877-5p inhibitor.

Additionally, the qRT-PCR and WB results showed that FOXM1 mRNA and protein were greatly up-regulated in the AMC-HN-8 cells in the presence of pcDNA3.1-hsa_circ_0042823. Overexpression of miR-877-5p notably repressed the mRNA and protein of FOXM1 in AMC-HN-8 cells. MiR-877-5p overexpression-mediated inhibition of FOXM1 expression was partially abrogated by hsa_circ_0042823 up-regulation in AMC-HN-8 cells (Figure 4(A,B)). In the TU686 cells, hsa_circ_0042823 silencing suppressed the mRNA and protein of FOXM1, while miR-877-5p deficiency caused an up-regulation of FOXM1 mRNA and protein. The influence conferred by miR-877-5p knockdown was abolished by up-regulation of hsa_circ_0042823 (Figure 4(C,D)).

Based on these findings, it can be considered that hsa_circ_0042823 functioned as a ceRNA to repress miR-877-5p, and then controlled the expression of FOXM1.

### Hsa_circ_0042823 overexpression regulated miR-877-5p/FOXM1 axis to promote proliferation, migration and invasion of AMC-HN-8 cells

**Figure 5.**
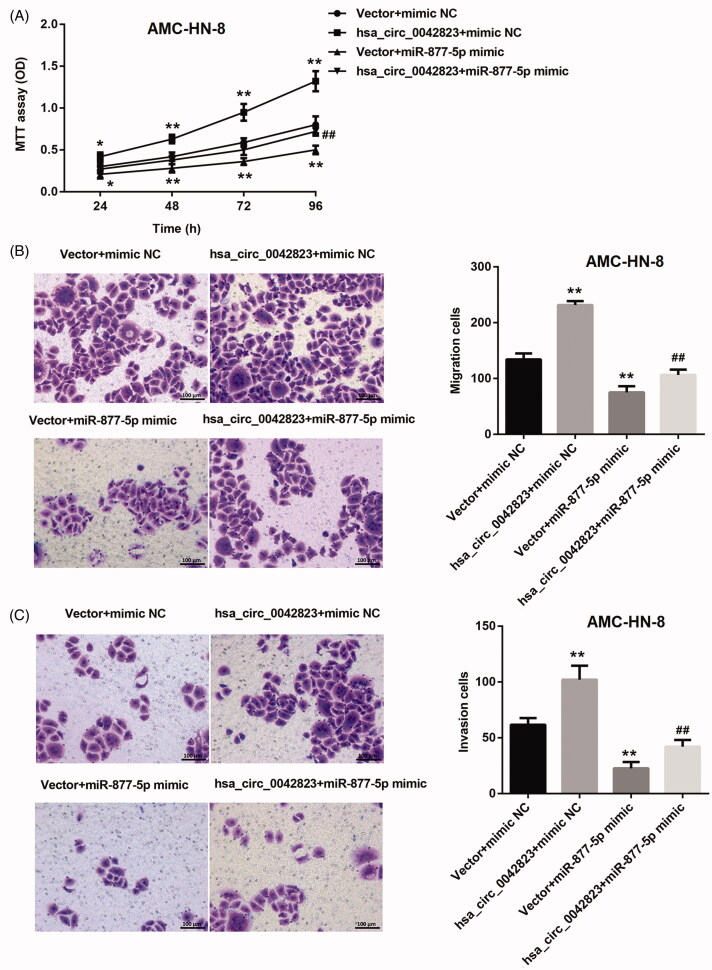
Hsa_circ_0042823 overexpression inhibited miR-877-5p to promote AMC-HN-8 cell proliferation, migration and invasion. (A) MTT assay was performed to explore proliferation of the AMC-HN-8 cells following up-regulation of hsa_circ_0042823 combined with miR-877-5p. (B and C) Transwell assay was performed to detect migration and invasion of the AMC-HN-8 cells following up-regulation of hsa_circ_0042823 combined with miR-877-5p. **p<.01 compared with Vector + mimic NC; ^##^*p*<.01 compared with Vector + miR-877-5p mimic.

Here, we determined whether hsa_circ_0042823/miR-877-5p/FOXM1 axis affected cell proliferation, migration and invasion in LSCC. The growth curves obtained from MTT assay showed that hsa_circ_0042823 overexpression significantly promoted the ability of cell proliferation in AMC-HN-8 cells. However, miR-877-5p overexpression repressed AMC-HN-8 cell proliferation, which was partly rescued by up-regulation of hsa_circ_0042823 (Figure 5(A)). Moreover, the results of transwell assay confirmed that the ability of migration and invasion of AMC-HN-8 cells was significantly enhanced by up-regulation of hsa_circ_0042823. AMC-HN-8 cell migration and invasion were severely suppressed by overexpression of miR-877-5p. The decrease of migration and invasion of AMC-HN-8 cells caused by the transfection of miR-877-5p mimic was effectively rescued by up-regulation of hsa_circ_0042823 (Figure 5(B,C)).

**Figure 6.**
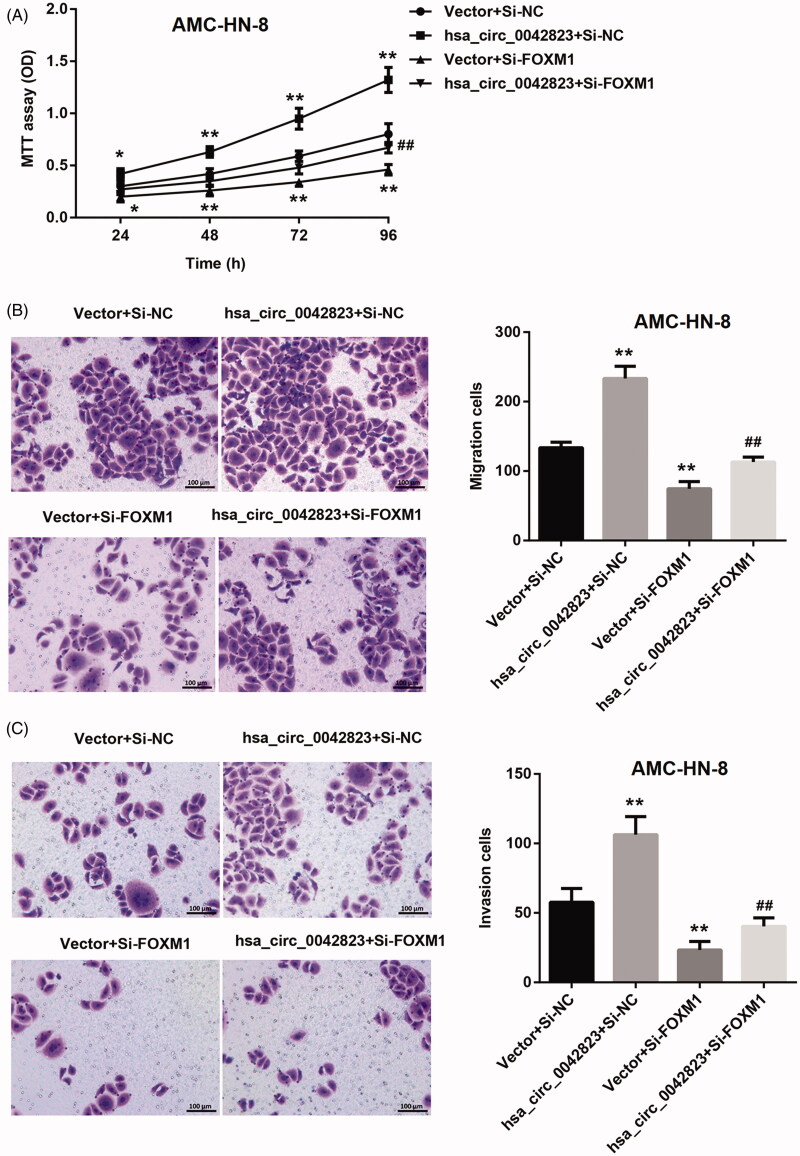
Hsa_circ_0042823 overexpression promoted FOXM1 to enhance AMC-HN-8 cell proliferation, migration and invasion. (A) MTT assay was performed to explore proliferation of the modified AMC-HN-8 cells following hsa_circ_0042823 overexpression combined with FOXM1 knockdown. (B and C) Transwell assay was performed to detect migration and invasion of the AMC-HN-8 cells following hsa_circ_0042823 overexpression combined with FOXM1 knockdown. **p<.01 compared with Vector + Si-NC; ^##^*p*<.01 compared with Vector + Si-FOXM1.

Next, the results of MTT assay confirmed the promoting effect of hsa_circ_0042823 up-regulation on proliferation of AMC-HN-8 cells. FOXM1 deficiency severely suppressed proliferation of AMC-HN-8 cells. FOXM1 knockdown-mediated inhibition of AMC-HN-8 cell proliferation was abolished by hsa_circ_0042823 overexpression (Figure 6(A)). Moreover, hsa_circ_0042823 overexpression led to a boost in migration and invasion of AMC-HN-8 cells. Inhibition of FOXM1 inhibited the ability of migration and invasion inAMC-HN-8 cells. The inhibiting effect of FOXM1 deficiency on AMC-HN-8 cell migration and invasion was partly abolished by up-regulation of hsa_circ_0042823 (Figure 6(B,C)).

Taken together, hsa_circ_0042823/miR-877-5p/FOXM1 axis regulated proliferation, migration and invasion of AMC-HN-8 cells.

### Hsa_circ_0042823 knockdown inhibited tumour growth of LSCC through regulating miR-877-5p/FOXM1 axis

**Figure 7.**
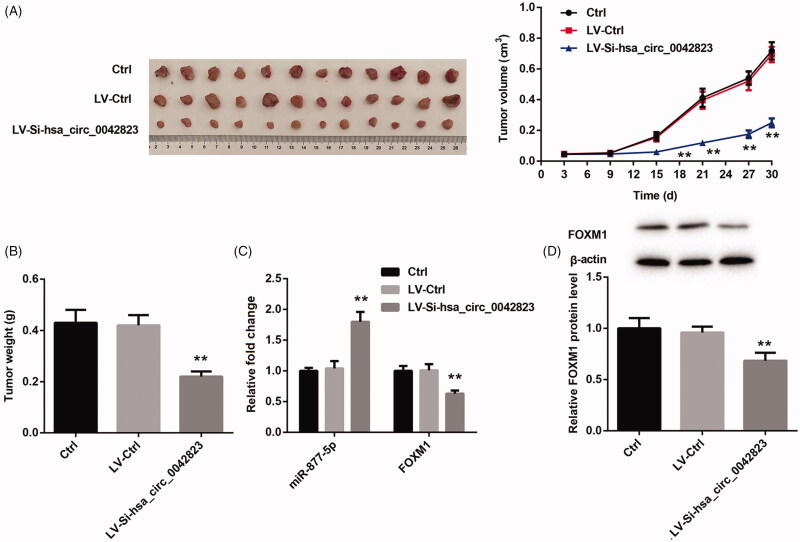
Hsa_circ_0042823 knockdown inhibited the tumour growth of LSCC *via* miR-877-5p/FOXM1 axis. (A and B) The tumour volume and weight in Ctrl, LV-Ctrl and LV-Si-hsa_circ_0042823 groups was measured. (C and D) The mRNA or protein expression of miR-877-5p and FOXM1 in Ctrl, LV-Ctrl and LV-Si-hsa_circ_0042823 groups was estimated by qRT-PCR and WB. **p<.01, compared with LV-Ctrl.

Finally, we verified whether hsa_circ_0042823 affected tumour growth of LSCC *via* miR-877-5p/FOXM1 axis. We used hsa_circ_0042823-silenced AMC-HN-8 cells to construct tumour xenografted mouse model, and observed the tumour growth of LSCC. Figure 7(A,B) showed that LV-si-hsa_circ_0042823 notably repressed the volume and weight of tumour tissues in mice. Subsequently, we estimated miR-877-5p and FOXM1 expression in the tumour tissues by qRT-PCR and WB. The tumour tissues in LV-si-hsa_circ_0042823 group exhibited an increase of miR-877-5p expression and a decrease in the mRNA and protein expression of FOXM1 (Figure (C,D)). Therefore, these data confirmed that hsa_circ_0042823 knockdown inhibited the tumour growth of LSCC *via* miR-877-5p/FOXM1 axis.

## Discussion

CircRNAs have the function of participating in post-transcriptional regulation and binding to RNA-binding proteins. CircRNAs target and bind to the endogenous microRNA (miRNA), and inhibit the biological activity of miRNA. CircRNAs have become a current research hotspot in tumours [4]. Xuan et al. have used microarray analysis and confirmed that there are a total of 698 abnormally expressed circRNAs in the tumour tissues of LSCC, among which hsa_circRNA_100855 and hsa_circRNA_104912 have the most significant fold changes [10]. Hsa_circRNA_100855 and hsa_circRNA_104912 are related to tumour staging, lymph node metastasis, and clinical staging. CircRNA_103862 is highly expressed in the tumour tissues of LSCC. CircRNA_103862 regulates the expression of GOLM1 by interacting with miR-493-5p, and thus promotes proliferation and invasion of LSCC cells [16]. CircCORO1C acts as a sponge of let-7c-5p, and regulates its downstream gene PBX3 in LSCC, and CircCORO1C/let-7c-5p/PBX3 axis affects LSCC progression [17]. In this study, we first confirmed the role of hsa_circ_0042823 in LSCC. The expression of hsa_circ_0042823 was significantly increased in LSCC cells as compared with normal HBECs, indicating that hsa_circ_0042823 may be closely associated with the development of LSCC. Subsequently, we verified that hsa_circ_0042823 overexpression had a promoting effect on proliferation, migration and invasion of LSCC cells. Moreover, the inhibition of hsa_circ_0042823 notably repressed proliferation, migration and invasion of LSCC cells. Thus, these data suggested that hsa_circ_0042823 participated in the development of LSCC by affecting proliferation, migration and invasion of LSCC cells.

MiR-877-5p is a tumour suppressor that involved in the progression of various tumours. In cervical cancer, miR-877-5p acts as a downstream target of long non-coding RNA DSCAM-AS1, and DSCAM-AS1 plays a vital role in the tumorigenesis *via* miR-877-5p/ATXN7L3 axis in cervical cancer [18]. Hepatocellular carcinoma tissues and cell lines all display a low expression of miR-877-5p, and down-regulation of miR-877-5p predicts the prognosis in hepatocellular carcinoma [11]. Wang et al. have demonstrated that SNHG16/miR-877-5p/FOXP4 axis participates in LSCC development [19]. Here, we confirmed that miR-877-5p was the downstream target of hsa_circ_0042823. Wu et al. have found that FOXM1 is a downstream target gene of miR-877-5p in gastric cancer [20]. Moreover, we also demonstrated that miR-877-5p interacted with 3′ UTR FOXM1 in LSCC cells. Hsa_circ_0042823 overexpression increased the expression of FOXM1, while up-regulation of miR-877-5p repressed FOXM1 expression. MiR-877-5p up-regulation-mediated inhibition of FOXM1 expression was partly rescued by hsa_circ_0042823 overexpression. Moreover, miR-877-5p silencing enhanced the mRNA and protein expression of FOXM1, which was partly abolished by hsa_circ_0042823 up-regulation. Thus, hsa_circ_0042823 promoted the expression of FOXM1 by competitively binding miR-877-5p.

FOXM1 is up-regulated in LSCC cells. FOXM1 overexpression may be a useful prognostic marker for LSCC [21]. Thiostrepton has an ability to reduce cell proliferation and enhance cell apoptosis by targeting FOXM1, thereby inhibiting tumorigenesis of LSCC in nude mice [15]. In LSCC, miR-370 exerts a tumour suppressor effect through inhibiting the expression of FoxM1 [22]. Our findings demonstrated that hsa_circ_0042823 overexpression enhanced LSCC cell proliferation, migration and invasion. The proliferation, migration and invasion of LSCC cells were repressed by miR-877-5p overexpression or FOXM1 knockdown. However, hsa_circ_0042823 up-regulation rescued miR-877-5p overexpression or FOXM1 knockdown-mediated inhibition of proliferation, migration and invasion of LSCC cells. Thus, hsa_circ_0042823 overexpression regulated miR-877-5p/FOXM1 axis to promote proliferation, migration and invasion of LSCC cells. Finally, our *in vivo* experiments revealed that inhibition of hsa_circ_0042823 notably reduced tumour growth in mouse model of LSCC through regulating miR-877-5p/FOXM1 axis.

In sum, our study confirms that hsa_circ_0042823 acts as an oncogenic circRNA in the progression of LSCC, and hsa_circ_0042823 promotes the expression of FOXM1 by competitively binding to miR-877-5p. Thus, hsa_circ_0042823 is potentially a novel target for LSCC treatment.

## Data Availability

The datasets in this study are available from the corresponding author on reasonable request.
